# Supplementary material for: End Sequence Analysis Toolkit (ESAT) expands the extractable information from single-cell RNA-seq data
Source: Genome Res. 2016 Oct;26(10):1397–410. doi: 10.1101/gr.207902.116 (PMC5052061; doi:10.1101/gr.207902.116)
Supplement: Supplemental Material [file supp_gr.207902.116_Supp_Materials.tar.gz › ESAT-esat_v0.1/doc/html/classbroad_1_1core_1_1math_1_1_statistics.html]

ESAT: broad.core.math.Statistics Class Reference


ESAT


- Main Page
- Related Pages
- Packages
- Classes
- Files

- Class List
- Class Index
- Class Hierarchy
- Class Members

All Classes Namespaces Files Functions Variables Enumerator Pages

- broad
- core
- math
- Statistics

Public Member Functions |
Static Public Member Functions |
Static Public Attributes |
List of all members

broad.core.math.Statistics Class Reference

|  |  |
| --- | --- |
| Public Member Functions | |
| double | manhattanDistance (double[] x, double[] y) |
|  | |

|  |  |
| --- | --- |
| Static Public Member Functions | |
| static double | median (Collection< Double > list) |
|  | |
| static double | median (double[] list) |
|  | |
| static double | median (int[] list) |
|  | |
| static double | quantile (List<?extends Number > list, double pct) |
|  | |
| static double | quantile (double[] vals, double pct) |
|  | |
| static double | mean (double[] values) |
|  | |
| static double | mean (Matrix data) |
|  | |
| static double | mean (Collection<?extends Number > values) |
|  | |
| static double | average (Collection<?extends Number > values) |
|  | |
| static double | average (double[] values) |
|  | |
| static double | variance (double[] values) |
|  | |
| static double | variance (double[] values, double mean) |
|  | |
| static double | variance (List<?extends Number > values) |
|  | |
| static double | variance (List<?extends Number > values, double mean) |
|  | |
| static double | covariance (List<?extends Number > values1, List<?extends Number > values2, double mean1, double mean2) |
|  | |
| static double | covariance (List<?extends Number > values1, List<?extends Number > values2) |
|  | |
| static double | pearsonCorrelation (List<?extends Number > values1, List<?extends Number > values2) |
|  | |
| static double | min (Collection< Double > vals) |
|  | |
| static double | max (Collection< Double > vals) |
|  | |
| static double | min (double[] vals) |
|  | |
| static double | max (double[] vals) |
|  | |
| static int | minInt (Collection< Integer > vals) |
|  | |
| static int | maxInt (Collection< Integer > vals) |
|  | |
| static int | min (int[] vals) |
|  | |
| static int | max (int[] vals) |
|  | |
| static double | metaAvg (Collection< double[]> data) |
|  | |
| static double | sum (List< Double > vals) |
|  | |
| static double | sum (double[] vals) |
|  | |
| static double | tstat (List< Double > g1, List< Double > g2, double tstat\_tuning\_param) |
|  | |
| static double | tstat (double[] g1, double[] g2, double tstat\_tuning\_param) |
|  | |
| static double | diff (double[] g1, double[] g2) |
|  | |
| static double | maxmean (double[] vals) |
|  | |
| static double | pairedTStat (List< Double > g1, List< Double > g2) |
|  | |
| static double | pairedTStat (double[] gr1, double[] gr2) |
|  | |
| static double | stdev (Collection<?extends Number > values) |
|  | |
| static double | stdev (double[] values) |
|  | |
| static double | pearsonDistance (double[] x, double[] y) |
|  | |
| static double | pearsonDistance (List< Double > xA, List< Double > yA) |
|  | |
| static double | spearmanCorrelation (List< Double > xA, List< Double > yA) |
|  | |
| static double | spearmanCorrelation (double[] x, double[] y) |
|  | |
| static double | euclideanDistance (double[] x, double[] y) |
|  | |
| static double | euclideanDistance (List< Double > x, List< Double > y) |
|  | |
| static double | FDRCorrect (double[] pvals, double alpha) |
|  | |
| static double | FDRCorrect (List< Double > pvals, double alpha) |
|  | |
| static double | FDR (EmpiricalDistribution observedDist, EmpiricalDistribution[] randomDist, double x) |
|  | |
| static double[] | hochbergPvalueAdjust (double[] pvals) |
|  | |
| static double | pvalue (List< Double > data, double value, boolean isSorted) |
|  | |
| static double | pvalue (double[] data, double value, boolean isSorted) |
|  | |
| static double | pvalue (List< Double > data, double value) |
|  | |
| static void | main (String[] args) throws Exception |
|  | |
| static double | zScore (double observation, double[] controlObservations, double fudge\_factor) |
|  | |
| static double | zScore (double observation, double mean, double variance, int length) |
|  | |
| static double | tstat (List< Double > g1, List< Double > g2) |
|  | |
| static double | zScore (double observation, double[] controlObservations) |
|  | |
| static double[] | computePermutedZScores (double observation, double[] controlObservations, double fudge\_factor) |
|  | |
| static double[] | computePermutedZScores (double observation, double[] controlObservations) |
|  | |
| static double[] | rank (double[] vals, boolean ascending) |
|  | |
| static double | absFold (Collection< Double > g1, Collection< Double > g2, boolean log) |
|  | |
| static double | absFold (double[] g1, double[] g2, boolean log) |
|  | |
| static double | fold (Collection< Double > g1, Collection< Double > g2, boolean log) |
|  | |
| static double | fold (double[] g1, double[] g2, boolean log) |
|  | |
| static double | fold (double[] g1, boolean log) |
|  | |
| static double | medianCollection (Collection< Double > values) |
|  | |
| static double | hypergeometric (int numberOfGenesTotal, int diffExpressedGenes, int genesInSet, int diffExpressedGenesInSet) |
|  | |
| static double | MAD (double[] array) |
|  | |
| static double | maxStdev (Matrix data) |
|  | |
| static double | maxMAD (Matrix data) |
|  | |
| static double | geometricMean (double[] vals) |
|  | |
| static double | geometricMeanMAD (Matrix data) |
|  | |
| static double | anovaFStat (Collection< double[]> lists) |
|  | |
| static int[] | randomPermutation (int n) |
|  | |
| static double[] | toDoubleArray (List< Double > lst) |
|  | |
| static ArrayList< Double > | toDoubleArrayList (double[] intervalScores) |
|  | |
| static ArrayList< Double > | toUniqueSortedList (ArrayList< Double > List) |
|  | |
| static Matrix | absoluteValue (Matrix permutations) |
|  | |
| static double[] | absoluteValue (double[] observed) |
|  | |
| static double | entropy (double[] p\_vec) |
|  | |
| static double | hamming (double[] x, double[] y, double threshold) |
|  | |
| static double | hamming (List< Double > x, List< Double > y, double threshold) |
|  | |
| static Double | JSDist (double[] p1, double[] p2) |
|  | |
| static double[] | normalizeToRelativeAbundance (double[] vals) |
|  | |
| static double | zscoreToPvalue (double zscore) |
|  | |
| static double | zScore2 (double observation, double[] controlObservations) |
|  | |
| static double | getKSStatistic (double[] observed, double[] expected) |
|  | |

|  |  |
| --- | --- |
| Static Public Attributes | |
| static final String | USAGE |
|  | |

## Member Function Documentation

|  |  |  |  |
| --- | --- | --- | --- |
| static double broad.core.math.Statistics.absFold | ( | Collection< Double > | *g1*, |
|  |  | Collection< Double > | *g2*, |
|  |  | boolean | *log* |
|  | ) |  |  |

static

Here is the call graph for this function:

|  |  |  |  |
| --- | --- | --- | --- |
| static double broad.core.math.Statistics.absFold | ( | double[] | *g1*, |
|  |  | double[] | *g2*, |
|  |  | boolean | *log* |
|  | ) |  |  |

static

Here is the call graph for this function:

|  |  |  |  |  |  |
| --- | --- | --- | --- | --- | --- |
| static Matrix broad.core.math.Statistics.absoluteValue | ( | Matrix | *permutations* | ) |  |

static

|  |  |  |  |  |  |
| --- | --- | --- | --- | --- | --- |
| static double [] broad.core.math.Statistics.absoluteValue | ( | double[] | *observed* | ) |  |

static

|  |  |  |  |  |  |
| --- | --- | --- | --- | --- | --- |
| static double broad.core.math.Statistics.anovaFStat | ( | Collection< double[]> | *lists* | ) |  |

static

|  |  |  |  |  |  |
| --- | --- | --- | --- | --- | --- |
| static double broad.core.math.Statistics.average | ( | Collection<?extends Number > | *values* | ) |  |

static

Here is the call graph for this function:

Here is the caller graph for this function:

|  |  |  |  |  |  |
| --- | --- | --- | --- | --- | --- |
| static double broad.core.math.Statistics.average | ( | double[] | *values* | ) |  |

static

Here is the call graph for this function:

|  |  |  |  |
| --- | --- | --- | --- |
| static double [] broad.core.math.Statistics.computePermutedZScores | ( | double | *observation*, |
|  |  | double[] | *controlObservations*, |
|  |  | double | *fudge\_factor* |
|  | ) |  |  |

static

Here is the call graph for this function:

Here is the caller graph for this function:

|  |  |  |  |
| --- | --- | --- | --- |
| static double [] broad.core.math.Statistics.computePermutedZScores | ( | double | *observation*, |
|  |  | double[] | *controlObservations* |
|  | ) |  |  |

static

Here is the call graph for this function:

|  |  |  |  |
| --- | --- | --- | --- |
| static double broad.core.math.Statistics.covariance | ( | List<?extends Number > | *values1*, |
|  |  | List<?extends Number > | *values2*, |
|  |  | double | *mean1*, |
|  |  | double | *mean2* |
|  | ) |  |  |

static

Here is the call graph for this function:

Here is the caller graph for this function:

|  |  |  |  |
| --- | --- | --- | --- |
| static double broad.core.math.Statistics.covariance | ( | List<?extends Number > | *values1*, |
|  |  | List<?extends Number > | *values2* |
|  | ) |  |  |

static

Here is the call graph for this function:

|  |  |  |  |
| --- | --- | --- | --- |
| static double broad.core.math.Statistics.diff | ( | double[] | *g1*, |
|  |  | double[] | *g2* |
|  | ) |  |  |

static

Here is the call graph for this function:

|  |  |  |  |  |  |
| --- | --- | --- | --- | --- | --- |
| static double broad.core.math.Statistics.entropy | ( | double[] | *p\_vec* | ) |  |

static

|  |  |  |  |
| --- | --- | --- | --- |
| static double broad.core.math.Statistics.euclideanDistance | ( | double[] | *x*, |
|  |  | double[] | *y* |
|  | ) |  |  |

static

Here is the call graph for this function:

Here is the caller graph for this function:

|  |  |  |  |
| --- | --- | --- | --- |
| static double broad.core.math.Statistics.euclideanDistance | ( | List< Double > | *x*, |
|  |  | List< Double > | *y* |
|  | ) |  |  |

static

Here is the call graph for this function:

|  |  |  |  |
| --- | --- | --- | --- |
| static double broad.core.math.Statistics.FDR | ( | EmpiricalDistribution | *observedDist*, |
|  |  | EmpiricalDistribution[] | *randomDist*, |
|  |  | double | *x* |
|  | ) |  |  |

static

Here is the call graph for this function:

|  |  |  |  |
| --- | --- | --- | --- |
| static double broad.core.math.Statistics.FDRCorrect | ( | double[] | *pvals*, |
|  |  | double | *alpha* |
|  | ) |  |  |

static

Here is the caller graph for this function:

|  |  |  |  |
| --- | --- | --- | --- |
| static double broad.core.math.Statistics.FDRCorrect | ( | List< Double > | *pvals*, |
|  |  | double | *alpha* |
|  | ) |  |  |

static

|  |  |  |  |
| --- | --- | --- | --- |
| static double broad.core.math.Statistics.fold | ( | Collection< Double > | *g1*, |
|  |  | Collection< Double > | *g2*, |
|  |  | boolean | *log* |
|  | ) |  |  |

static

Here is the call graph for this function:

Here is the caller graph for this function:

|  |  |  |  |
| --- | --- | --- | --- |
| static double broad.core.math.Statistics.fold | ( | double[] | *g1*, |
|  |  | double[] | *g2*, |
|  |  | boolean | *log* |
|  | ) |  |  |

static

Here is the call graph for this function:

|  |  |  |  |
| --- | --- | --- | --- |
| static double broad.core.math.Statistics.fold | ( | double[] | *g1*, |
|  |  | boolean | *log* |
|  | ) |  |  |

static

Here is the call graph for this function:

|  |  |  |  |  |  |
| --- | --- | --- | --- | --- | --- |
| static double broad.core.math.Statistics.geometricMean | ( | double[] | *vals* | ) |  |

static

Here is the call graph for this function:

|  |  |  |  |  |  |
| --- | --- | --- | --- | --- | --- |
| static double broad.core.math.Statistics.geometricMeanMAD | ( | Matrix | *data* | ) |  |

static

Here is the call graph for this function:

|  |  |  |  |
| --- | --- | --- | --- |
| static double broad.core.math.Statistics.getKSStatistic | ( | double[] | *observed*, |
|  |  | double[] | *expected* |
|  | ) |  |  |

static

|  |  |  |  |
| --- | --- | --- | --- |
| static double broad.core.math.Statistics.hamming | ( | double[] | *x*, |
|  |  | double[] | *y*, |
|  |  | double | *threshold* |
|  | ) |  |  |

static

computes the hamming distance between two vectors, given a threshold. The threshold is used to reduce the vectors to a trinary form were, for example x[i] is set to 1 when x[i]>threshold, x[i] is set to -1 when x[i]< threshold and 0 otherwise

Parameters
:   |  |  |
    | --- | --- |
    | x |  |
    | y |  |
    | threshold | to use in order to reduce the vectors |

Returns

Here is the caller graph for this function:

|  |  |  |  |
| --- | --- | --- | --- |
| static double broad.core.math.Statistics.hamming | ( | List< Double > | *x*, |
|  |  | List< Double > | *y*, |
|  |  | double | *threshold* |
|  | ) |  |  |

static

computes the hamming distance between two vectors, given a threshold. The threshold is used to reduce the vectors to a trinary form were, for example x.get(i) is set to 1 when x.get(i)>threshold, x.get(i)is set to -1 when x.get(i)< threshold and 0 otherwise

Parameters
:   |  |  |
    | --- | --- |
    | x |  |
    | y |  |
    | threshold | to use in order to reduce the vectors |

Returns

Here is the call graph for this function:

|  |  |  |  |  |  |
| --- | --- | --- | --- | --- | --- |
| static double [] broad.core.math.Statistics.hochbergPvalueAdjust | ( | double[] | *pvals* | ) |  |

static

Adjust a set of P values for multiple testing with the Hochberg method

Parameters
:   |  |  |
    | --- | --- |
    | pvals | The P values |

Returns
:   Array of adjusted P values in same order

|  |  |  |  |
| --- | --- | --- | --- |
| static double broad.core.math.Statistics.hypergeometric | ( | int | *numberOfGenesTotal*, |
|  |  | int | *diffExpressedGenes*, |
|  |  | int | *genesInSet*, |
|  |  | int | *diffExpressedGenesInSet* |
|  | ) |  |  |

static

Here is the call graph for this function:

|  |  |  |  |
| --- | --- | --- | --- |
| static Double broad.core.math.Statistics.JSDist | ( | double[] | *p1*, |
|  |  | double[] | *p2* |
|  | ) |  |  |

static

Here is the caller graph for this function:

|  |  |  |  |  |  |
| --- | --- | --- | --- | --- | --- |
| static double broad.core.math.Statistics.MAD | ( | double[] | *array* | ) |  |

static

Here is the call graph for this function:

Here is the caller graph for this function:

|  |  |  |  |  |  |
| --- | --- | --- | --- | --- | --- |
| static void broad.core.math.Statistics.main | ( | String[] | *args* | ) | throws Exception |

static

Here is the call graph for this function:

|  |  |  |  |
| --- | --- | --- | --- |
| double broad.core.math.Statistics.manhattanDistance | ( | double[] | *x*, |
|  |  | double[] | *y* |
|  | ) |  |  |

Here is the call graph for this function:

|  |  |  |  |  |  |
| --- | --- | --- | --- | --- | --- |
| static double broad.core.math.Statistics.max | ( | Collection< Double > | *vals* | ) |  |

static

Here is the caller graph for this function:

|  |  |  |  |  |  |
| --- | --- | --- | --- | --- | --- |
| static double broad.core.math.Statistics.max | ( | double[] | *vals* | ) |  |

static

Here is the call graph for this function:

|  |  |  |  |  |  |
| --- | --- | --- | --- | --- | --- |
| static int broad.core.math.Statistics.max | ( | int[] | *vals* | ) |  |

static

Here is the call graph for this function:

|  |  |  |  |  |  |
| --- | --- | --- | --- | --- | --- |
| static int broad.core.math.Statistics.maxInt | ( | Collection< Integer > | *vals* | ) |  |

static

Here is the call graph for this function:

|  |  |  |  |  |  |
| --- | --- | --- | --- | --- | --- |
| static double broad.core.math.Statistics.maxMAD | ( | Matrix | *data* | ) |  |

static

Here is the call graph for this function:

|  |  |  |  |  |  |
| --- | --- | --- | --- | --- | --- |
| static double broad.core.math.Statistics.maxmean | ( | double[] | *vals* | ) |  |

static

|  |  |  |  |  |  |
| --- | --- | --- | --- | --- | --- |
| static double broad.core.math.Statistics.maxStdev | ( | Matrix | *data* | ) |  |

static

Here is the call graph for this function:

|  |  |  |  |  |  |
| --- | --- | --- | --- | --- | --- |
| static double broad.core.math.Statistics.mean | ( | double[] | *values* | ) |  |

static

Here is the caller graph for this function:

|  |  |  |  |  |  |
| --- | --- | --- | --- | --- | --- |
| static double broad.core.math.Statistics.mean | ( | Matrix | *data* | ) |  |

static

Here is the call graph for this function:

|  |  |  |  |  |  |
| --- | --- | --- | --- | --- | --- |
| static double broad.core.math.Statistics.mean | ( | Collection<?extends Number > | *values* | ) |  |

static

|  |  |  |  |  |  |
| --- | --- | --- | --- | --- | --- |
| static double broad.core.math.Statistics.median | ( | Collection< Double > | *list* | ) |  |

static

Here is the caller graph for this function:

|  |  |  |  |  |  |
| --- | --- | --- | --- | --- | --- |
| static double broad.core.math.Statistics.median | ( | double[] | *list* | ) |  |

static

Here is the call graph for this function:

|  |  |  |  |  |  |
| --- | --- | --- | --- | --- | --- |
| static double broad.core.math.Statistics.median | ( | int[] | *list* | ) |  |

static

Here is the call graph for this function:

|  |  |  |  |  |  |
| --- | --- | --- | --- | --- | --- |
| static double broad.core.math.Statistics.medianCollection | ( | Collection< Double > | *values* | ) |  |

static

Here is the call graph for this function:

|  |  |  |  |  |  |
| --- | --- | --- | --- | --- | --- |
| static double broad.core.math.Statistics.metaAvg | ( | Collection< double[]> | *data* | ) |  |

static

Here is the call graph for this function:

|  |  |  |  |  |  |
| --- | --- | --- | --- | --- | --- |
| static double broad.core.math.Statistics.min | ( | Collection< Double > | *vals* | ) |  |

static

Here is the caller graph for this function:

|  |  |  |  |  |  |
| --- | --- | --- | --- | --- | --- |
| static double broad.core.math.Statistics.min | ( | double[] | *vals* | ) |  |

static

Here is the call graph for this function:

|  |  |  |  |  |  |
| --- | --- | --- | --- | --- | --- |
| static int broad.core.math.Statistics.min | ( | int[] | *vals* | ) |  |

static

Here is the call graph for this function:

|  |  |  |  |  |  |
| --- | --- | --- | --- | --- | --- |
| static int broad.core.math.Statistics.minInt | ( | Collection< Integer > | *vals* | ) |  |

static

Here is the call graph for this function:

|  |  |  |  |  |  |
| --- | --- | --- | --- | --- | --- |
| static double [] broad.core.math.Statistics.normalizeToRelativeAbundance | ( | double[] | *vals* | ) |  |

static

Here is the call graph for this function:

|  |  |  |  |
| --- | --- | --- | --- |
| static double broad.core.math.Statistics.pairedTStat | ( | List< Double > | *g1*, |
|  |  | List< Double > | *g2* |
|  | ) |  |  |

static

Here is the call graph for this function:

|  |  |  |  |
| --- | --- | --- | --- |
| static double broad.core.math.Statistics.pairedTStat | ( | double[] | *gr1*, |
|  |  | double[] | *gr2* |
|  | ) |  |  |

static

Here is the call graph for this function:

|  |  |  |  |
| --- | --- | --- | --- |
| static double broad.core.math.Statistics.pearsonCorrelation | ( | List<?extends Number > | *values1*, |
|  |  | List<?extends Number > | *values2* |
|  | ) |  |  |

static

Here is the call graph for this function:

|  |  |  |  |
| --- | --- | --- | --- |
| static double broad.core.math.Statistics.pearsonDistance | ( | double[] | *x*, |
|  |  | double[] | *y* |
|  | ) |  |  |

static

Here is the call graph for this function:

Here is the caller graph for this function:

|  |  |  |  |
| --- | --- | --- | --- |
| static double broad.core.math.Statistics.pearsonDistance | ( | List< Double > | *xA*, |
|  |  | List< Double > | *yA* |
|  | ) |  |  |

static

Here is the call graph for this function:

|  |  |  |  |
| --- | --- | --- | --- |
| static double broad.core.math.Statistics.pvalue | ( | List< Double > | *data*, |
|  |  | double | *value*, |
|  |  | boolean | *isSorted* |
|  | ) |  |  |

static

Computes what is P(X > value) using list of values

Parameters
:   |  |  |
    | --- | --- |
    | data |  |
    | value |  |
    | isSorted | true if the data list is sorted, it will avoid sorting here. |

Returns

Here is the caller graph for this function:

|  |  |  |  |
| --- | --- | --- | --- |
| static double broad.core.math.Statistics.pvalue | ( | double[] | *data*, |
|  |  | double | *value*, |
|  |  | boolean | *isSorted* |
|  | ) |  |  |

static

Computes what is P(X > value) using list of values

Parameters
:   |  |  |
    | --- | --- |
    | data |  |
    | value |  |
    | isSorted | true if the data list is sorted, it will avoid sorting here. |

Returns

|  |  |  |  |
| --- | --- | --- | --- |
| static double broad.core.math.Statistics.pvalue | ( | List< Double > | *data*, |
|  |  | double | *value* |
|  | ) |  |  |

static

Computes what is P(X > value) using list of values

Parameters
:   |  |  |
    | --- | --- |
    | data |  |
    | value |  |

Returns

Here is the call graph for this function:

|  |  |  |  |
| --- | --- | --- | --- |
| static double broad.core.math.Statistics.quantile | ( | List<?extends Number > | *list*, |
|  |  | double | *pct* |
|  | ) |  |  |

static

Uses the Weighted average method.

Parameters
:   |  |  |
    | --- | --- |
    | list | - Ordered list of numbers (i.e. short, float, double, etc) |
    | pct | - Desired quantile |

Returns
:   the estimated quantile requested: x s.t. P(X <= x) >= pct

|  |  |  |  |
| --- | --- | --- | --- |
| static double broad.core.math.Statistics.quantile | ( | double[] | *vals*, |
|  |  | double | *pct* |
|  | ) |  |  |

static

|  |  |  |  |  |  |
| --- | --- | --- | --- | --- | --- |
| static int [] broad.core.math.Statistics.randomPermutation | ( | int | *n* | ) |  |

static

|  |  |  |  |
| --- | --- | --- | --- |
| static double [] broad.core.math.Statistics.rank | ( | double[] | *vals*, |
|  |  | boolean | *ascending* |
|  | ) |  |  |

static

Here is the call graph for this function:

Here is the caller graph for this function:

|  |  |  |  |
| --- | --- | --- | --- |
| static double broad.core.math.Statistics.spearmanCorrelation | ( | List< Double > | *xA*, |
|  |  | List< Double > | *yA* |
|  | ) |  |  |

static

Here is the call graph for this function:

|  |  |  |  |
| --- | --- | --- | --- |
| static double broad.core.math.Statistics.spearmanCorrelation | ( | double[] | *x*, |
|  |  | double[] | *y* |
|  | ) |  |  |

static

Here is the call graph for this function:

|  |  |  |  |  |  |
| --- | --- | --- | --- | --- | --- |
| static double broad.core.math.Statistics.stdev | ( | Collection<?extends Number > | *values* | ) |  |

static

Here is the call graph for this function:

Here is the caller graph for this function:

|  |  |  |  |  |  |
| --- | --- | --- | --- | --- | --- |
| static double broad.core.math.Statistics.stdev | ( | double[] | *values* | ) |  |

static

Here is the call graph for this function:

|  |  |  |  |  |  |
| --- | --- | --- | --- | --- | --- |
| static double broad.core.math.Statistics.sum | ( | List< Double > | *vals* | ) |  |

static

Here is the caller graph for this function:

|  |  |  |  |  |  |
| --- | --- | --- | --- | --- | --- |
| static double broad.core.math.Statistics.sum | ( | double[] | *vals* | ) |  |

static

|  |  |  |  |  |  |
| --- | --- | --- | --- | --- | --- |
| static double [] broad.core.math.Statistics.toDoubleArray | ( | List< Double > | *lst* | ) |  |

static

|  |  |  |  |  |  |
| --- | --- | --- | --- | --- | --- |
| static ArrayList<Double> broad.core.math.Statistics.toDoubleArrayList | ( | double[] | *intervalScores* | ) |  |

static

|  |  |  |  |  |  |
| --- | --- | --- | --- | --- | --- |
| static ArrayList<Double> broad.core.math.Statistics.toUniqueSortedList | ( | ArrayList< Double > | *List* | ) |  |

static

|  |  |  |  |
| --- | --- | --- | --- |
| static double broad.core.math.Statistics.tstat | ( | List< Double > | *g1*, |
|  |  | List< Double > | *g2*, |
|  |  | double | *tstat\_tuning\_param* |
|  | ) |  |  |

static

Here is the call graph for this function:

Here is the caller graph for this function:

|  |  |  |  |
| --- | --- | --- | --- |
| static double broad.core.math.Statistics.tstat | ( | double[] | *g1*, |
|  |  | double[] | *g2*, |
|  |  | double | *tstat\_tuning\_param* |
|  | ) |  |  |

static

Here is the call graph for this function:

|  |  |  |  |
| --- | --- | --- | --- |
| static double broad.core.math.Statistics.tstat | ( | List< Double > | *g1*, |
|  |  | List< Double > | *g2* |
|  | ) |  |  |

static

Two sample T statistic

Parameters
:   |  |  |
    | --- | --- |
    | g1 | Measurements from group 1 |
    | g2 | Measurements from group 2 |

Returns
:   T statistic (positive iff mean of group 1 is greater than mean of group 2)

Here is the call graph for this function:

|  |  |  |  |  |  |
| --- | --- | --- | --- | --- | --- |
| static double broad.core.math.Statistics.variance | ( | double[] | *values* | ) |  |

static

Here is the call graph for this function:

Here is the caller graph for this function:

|  |  |  |  |
| --- | --- | --- | --- |
| static double broad.core.math.Statistics.variance | ( | double[] | *values*, |
|  |  | double | *mean* |
|  | ) |  |  |

static

Here is the call graph for this function:

|  |  |  |  |  |  |
| --- | --- | --- | --- | --- | --- |
| static double broad.core.math.Statistics.variance | ( | List<?extends Number > | *values* | ) |  |

static

Here is the call graph for this function:

|  |  |  |  |
| --- | --- | --- | --- |
| static double broad.core.math.Statistics.variance | ( | List<?extends Number > | *values*, |
|  |  | double | *mean* |
|  | ) |  |  |

static

Here is the call graph for this function:

|  |  |  |  |
| --- | --- | --- | --- |
| static double broad.core.math.Statistics.zScore | ( | double | *observation*, |
|  |  | double[] | *controlObservations*, |
|  |  | double | *fudge\_factor* |
|  | ) |  |  |

static

Here is the call graph for this function:

Here is the caller graph for this function:

|  |  |  |  |
| --- | --- | --- | --- |
| static double broad.core.math.Statistics.zScore | ( | double | *observation*, |
|  |  | double | *mean*, |
|  |  | double | *variance*, |
|  |  | int | *length* |
|  | ) |  |  |

static

|  |  |  |  |
| --- | --- | --- | --- |
| static double broad.core.math.Statistics.zScore | ( | double | *observation*, |
|  |  | double[] | *controlObservations* |
|  | ) |  |  |

static

Here is the call graph for this function:

|  |  |  |  |
| --- | --- | --- | --- |
| static double broad.core.math.Statistics.zScore2 | ( | double | *observation*, |
|  |  | double[] | *controlObservations* |
|  | ) |  |  |

static

Here is the call graph for this function:

|  |  |  |  |  |  |
| --- | --- | --- | --- | --- | --- |
| static double broad.core.math.Statistics.zscoreToPvalue | ( | double | *zscore* | ) |  |

static

Calculate p-value given a Z-score

Returns
:   double p-value

## Member Data Documentation

final String broad.core.math.Statistics.USAGE

static

**Initial value:**

= "Usage: Statistics TASK=<task\_num> <task\_args>\n" +

"\tTasks:\n" +

"\t\t1. Compute Benjamini-Hochberg FDR adjusted pvalue of a given pvalue column in a file " +

"\n\t\t\t-in <file (standard input is default)> -fdr <Maximum false discovery rate> -col <Data column, 0 based> [-separator <character used to separate columns, default is tab '\\t>'] " +

"\n"

---

The documentation for this class was generated from the following file:

- java/broad/core/math/Statistics.java


---

Generated on Fri Aug 15 2014 15:10:53 for ESAT by  

 1.8.7
